# Should Studies of Diabetes Treatment Stratification Correct for Baseline HbA1c?

**DOI:** 10.1371/journal.pone.0152428

**Published:** 2016-04-06

**Authors:** Angus G. Jones, Mike Lonergan, William E. Henley, Ewan R. Pearson, Andrew T. Hattersley, Beverley M. Shields

**Affiliations:** 1 NIHR Exeter Clinical Research Facility, University of Exeter Medical School, Exeter, United Kingdom; 2 Medical Research Institute, University of Dundee, Dundee, United Kingdom; 3 Institute of Health Services Research, University of Exeter Medical School, Exeter, United Kingdom; University of North Carolina at Chapel Hill, UNITED STATES

## Abstract

**Aims:**

Baseline HbA1c is a major predictor of response to glucose lowering therapy and therefore a potential confounder in studies aiming to identify other predictors. However, baseline adjustment may introduce error if the association between baseline HbA1c and response is substantially due to measurement error and regression to the mean. We aimed to determine whether studies of predictors of response should adjust for baseline HbA1c.

**Methods:**

We assessed the relationship between baseline HbA1c and glycaemic response in 257 participants treated with GLP-1R agonists and assessed whether it reflected measurement error and regression to the mean using duplicate ‘pre-baseline’ HbA1c measurements not included in the response variable. In this cohort and an additional 2659 participants treated with sulfonylureas we assessed the relationship between covariates associated with baseline HbA1c and treatment response with and without baseline adjustment, and with a bias correction using pre-baseline HbA1c to adjust for the effects of error in baseline HbA1c.

**Results:**

Baseline HbA1c was a major predictor of response (R^2^ = 0.19,β = -0.44,p<0.001).The association between pre-baseline and response was similar suggesting the greater response at higher baseline HbA1cs is not mainly due to measurement error and subsequent regression to the mean. In unadjusted analysis in both cohorts, factors associated with baseline HbA1c were associated with response, however these associations were weak or absent after adjustment for baseline HbA1c. Bias correction did not substantially alter associations.

**Conclusions:**

Adjustment for the baseline HbA1c measurement is a simple and effective way to reduce bias in studies of predictors of response to glucose lowering therapy.

## Introduction

Baseline HbA1c is a major predictor of glycaemic response to all glucose lowering therapies, accounting for up to 40% of individual variation in response [1]. Patients with a high baseline HbA1c have a greater reduction with treatment than those with a low baseline, even for non-pharmacological interventions [1–5].

As many clinical characteristics and biomarkers may be associated with baseline glycaemia, adjusting for this potential confounder in analyses of treatment response may be important in order to avoid erroneous results. Failure to account for baseline HbA1c may lead to false associations between a potential predictor and response, which are solely a result of it being positively associated with baseline HbA1c, or a true predictor which has an inverse relationship with baseline glycaemia may be missed.

Many existing studies of stratified medicine in Type 2 diabetes have adjusted for baseline glycaemia using regression based methods [6, 7]. However, this may increase rather than reduce bias if an association between baseline and response is wholly or partly due to random error in the baseline HbA1c measurement, leading to regression to the mean [8–12]. A high baseline measurement is more likely to have upwards random variation (i.e. the measured HbA1c is higher than the “true” HbA1c due to biological variation and/or analytical error), whereas a low measurement is more likely to be lower than the “true” HbA1c. These results would regress to the mean if repeated. The presence of this random error would exaggerate the estimated association between baseline HbA1c and change in HbA1c and potentially introduce bias into estimated effects of predictor variables [13]. For this reason, there have been concerns as to whether adjustment for baseline is appropriate even where an apparent relationship between baseline and response exists [14]. However, methods are available to assess the impact of measurement error and adjust for any potential bias in analysis by using repeat measurements of the baseline assessment [8].

There is currently no consistency in the use of baseline adjustment when assessing predictors of treatment response in diabetes. To our knowledge, the appropriateness of adjustment for HbA1c and the extent to which it affects results has not been previously assessed. We aimed to assess to what extent the relationship between baseline HbA1c and response to glucose lowering therapy is influenced by measurement error and ‘regression to the mean’, and whether adjustment for baseline HbA1c is appropriate when analysing potential predictors of diabetes treatment response.

## Patients and Methods

### Ethical approval

All participants in PRIBA and GoDARTs studies gave written informed consent. PRIBA was ethically approved by the South West Research Ethics committee (UK). The GoDARTS study was approved by the Tayside Medical Ethics Committee (UK)

### Study cohort

257 participants with a clinical diagnosis of type 2 diabetes and HbA1c ≥58mmol/mol (7.5%) commencing GLP-1 receptor agonist (GLP-1RA) therapy as part of their usual diabetes care prospectively recruited to the Predicting Response to Incretin Based Agents (PRIBA) study (https://clinicaltrials.gov/ct2/show/NCT01503112). HbA1c, glucose lowering therapy and adherence (self reported over previous two weeks) was assessed at baseline, 3 months (10–14 weeks) and 6 months (22–26 weeks) after commencing GLP-1 therapy. HbA1c measured in the 6 months prior to baseline (>1 to <26 weeks pre baseline, closest available clinical result) was also recorded where available (n = 153). All 257 participants included in this analysis were non insulin treated and completed >10 weeks GLP-1RA therapy without change in concurrent glucose lowering co-therapy (excluding dose changes, which were not associated with response (p = 0.3)). Treatment response (HbA1c change: on treatment value minus baseline) was calculated based on the latest available on-treatment HbA1c meeting these criteria. A study overview is given in **S1 Fig.**

### Analysis: to what extent is the association between baseline HbA1c and treatment response due to random error and regression to the mean?

In participants with HbA1c measured in the 6 months prior to baseline (n = 153) we assessed the relationship between baseline HbA1c and HbA1c change after treatment using Pearson’s correlation coefficients and linear regression analysis. We then performed the same analysis using HbA1c recorded prior to study commencement (‘pre-baseline HbA1c’) to assess whether this relationship between HbA1c and treatment response persisted when using an alternative pre-baseline HbA1c recorded on a separate occasion to the measurement included in the calculation of HbA1c change. Persistence of an association would suggest that the greater fall in HbA1c seen in those with higher baseline is not only due to random error in the baseline value and regression to the mean.

To further explore this question we examined the relationship between baseline HbA1c and the change between pre-baseline and baseline HbA1c values, using linear regression. If regression to the mean was contributing to the greater HbA1c fall after treatment seen in those with higher baseline glycaemia we would expect to see a greater increase in HbA1c from pre-baseline to baseline in those with higher HbA1c at study entry.

### Analysis: Is adjusting for baseline HbA1c appropriate when analysing potential predictors of diabetes treatment response?

To determine whether adjustment for baseline is appropriate in analysis of predictors of treatment response, we examined the relationship between 4 baseline variables (chosen to demonstrate a range of associations with study baseline HbA1c: fasting triglycerides, weight, creatinine, fasting glucose) and treatment response (HbA1c change at 6 months) in 3 ways:

1. The unadjusted association between baseline covariate and absolute glycaemic response (simple linear regression)
2. The association between the baseline covariate and treatment response after adjustment for baseline HbA1c (multiple linear regression)
3. The association between the baseline covariate and treatment response adjusted for study baseline HbA1c with a bias correction applied (Yanez method [8]).

The Yanez method [8] uses estimates of measurement error derived from the repeat baseline measurements and the associations between baseline and change to adjust for baseline error (and therefore regression to the mean) in the full dataset. If results are similar with and without the Yanez correction this would suggest that measurement error bias is limited; therefore standard regression methods and a single baseline measurement can be used.

In all cases baseline covariates were expressed as standard deviation scores, so the beta coefficients represented difference in HbA1c treatment response (in mmol/mol) for a 1 standard deviation increase in the baseline variable. This enables direct comparison across variables with different units of measurement.

### Analysis of baseline adjustment in a large Sulfonylurea treated cohort

We repeated analysis 1–3 above assessing the effect of adjusting for baseline HbA1c when studying predictors of treatment response in a cohort of response data from 2659 participants with type 2 diabetes treated with sulfonylurea therapy in the Genetics of Diabetes Audit and Research Tayside (GoDARTS) study cohort (http://diabetesgenetics.dundee.ac.uk/Default.aspx). All included participants had HbA1c measures between 3 and 9 months after commencing sulfonylurea therapy (outcome based on closest measure to 6 months), and had not changed glucose lowering co-treatments (excluding dose changes) at the time of on treatment HbA1c measurement. 1809 participants had duplicate HbA1c measurement in the 6 months prior to baseline (pre treatment) HbA1c, without change in glucose lowing co-therapy between these measurements. Fasting glucose was not available in this cohort and adherence (% of days between baseline and outcome HbA1c covered by encashed prescriptions [15]) was substituted for these measures.

### Assessment of use of change as percentage of baseline as a simple method of minimising confounding by baseline glycaemia

A simple method of reducing the effect of baseline glycaemia is to express change in HbA1c after treatment as a percentage of the baseline value. We therefore examined the relationship between baseline characteristics and glycaemic response as a percentage of baseline HbA1c for both cohorts using linear regression without baseline adjustment and compared this with the results of analysis 1–3 above.

### Laboratory analysis

HbA1c (PRIBA and GoDARTS) was measured in CPA {, #1010} accredited NHS laboratories standardised to IFCC reference method procedure {Sacks, 2005 #1011}, all repeated measurements within the same individual were analysed within the same laboratory using the same method. Other analysis for the PRIBA study was performed by the Biochemistry Department at the Royal Devon and Exeter Hospital, Exeter, UK. Triglycerides and creatinine in GoDARTS were measured by CPA accredited NHS laboratories as part of participants routine healthcare.

### Statistical analysis

Linear regression analysis and the Yanez bias correction calculations were performed using R version 3.1.2 (http://www.r-project.org/), with bootstrapping used to estimate the standard errors for the bias corrected beta-coefficients. Other analysis was carried out using Stata Statistical Software: Release 13 (StataCorp. 2013. College Station, TX).

## Results

### Participant characteristics

Participants’ characteristics (GLP-1R agonist cohort) are presented in **Table 1**. Pre-baseline HbA1c and baseline HbA1c were performed a median 8 weeks apart, were not significantly different (median 85 vs 84mmol/mol, p = 0.9) and were strongly associated (r = 0.81). Characteristics of the sulfonylurea cohort are shown in **S1 Table.**

**Table 1:** Participants baseline characteristics (GLP-1RA cohort, n = 257).

Baseline characteristics	Median (IQR) or %
Baseline HbA1c (mmol/mol)	84 (71–96)
Baseline HbA1c (%)	9.8 (8.6–10.9)
Pre-baseline HbA1c (mmol/mol)*	85 (70–96)
Pre-baseline HbA1c (%)	9.9 (8.6–10.9)
Time from pre-baseline to baseline HbA1c (days)*	57 (35–111)
HbA1c change (6months– 0 months, mmol/mol)	-18 (-30–-8)
HbA1c change (6months– 0 months, %)	-1.6 (-2.7–-0.7)
% Male	52%
Number of baseline OHAs	0 = 0.7%, 1 = 29%, 2 = 56%, 3 = 15%
Age (years)	55 (48–61)
Diabetes duration (years)	7 (4–11)
BMI (kg/m2)	40 (35–45)
Weight (kg)	113 (101–142)
Triglycerides (mmol/L)	2.1 (1.5–2.7)
Creatinine (umol/L)	71 (58–86)
Fasting glucose (mmol/L)	11.8 (9.7–14.5)

*n = 153

### Baseline HbA1c is a major predictor of response to glucose lowering therapy

**Fig 1:**
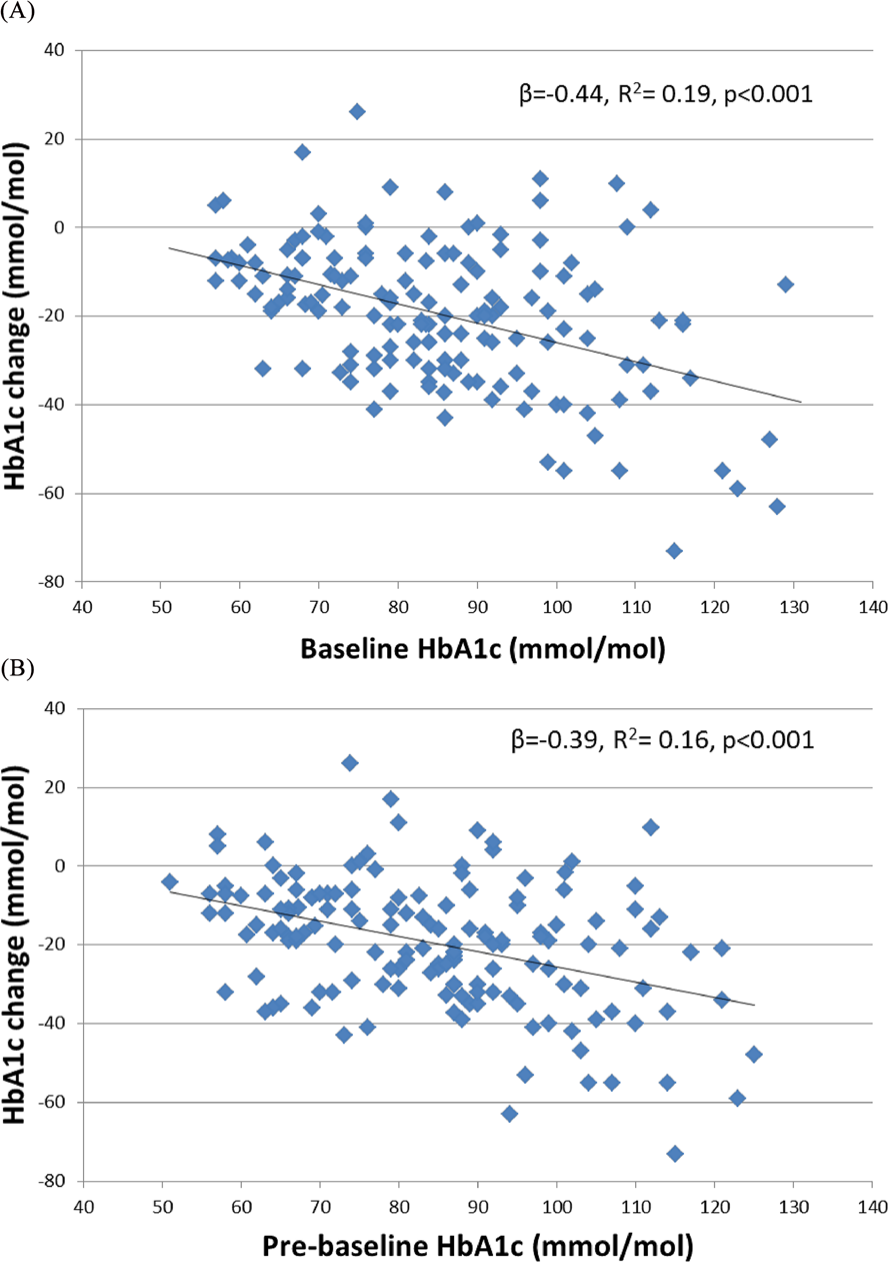
The association between post treatment HbA1c change and A: baseline HbA1c and B: Pre-baseline HbA1c (GLP-1RA cohort).

Baseline HbA1c explains 19% of variation in HbA1c change in GLP-1RA study participants (R^2^ = 0.19, p<0.0001, **Fig 1A**). A 1 mmol/mol higher baseline HbA1c is associated with a 0.4mmol/mol greater HbA1c reduction (linear regression β = -0.44mmol/mmol (95% CI -0.58, -0.29), p<0.001)). Therefore those with a high baseline HbA1c have a greater reduction in HbA1c compared with those with lower baseline values, but are still less likely to achieve glycaemic targets after treatment.

### The association between baseline HbA1c and change after treatment is not solely due to measurement error and regression to the mean

HbA1c change is associated with a person’s pre-treatment HbA1c even when pre-treatment HbA1c is measured on a separate occasion up to 6 months prior to the commencement of treatment (**Fig 1B**). Pre-baseline HbA1c explains 16% of GLP-1RA treatment response variation, and a 1mmol/mol increase in baseline HbA1c is association with a 0.4 mmol/mol greater reduction in HbA1c after treatment (R^2^ = 0.16, linear regression β = -0.39 (95% CI -0.52, -0.24), p<0.001). This result is similar to the association with baseline, suggesting that the association between baseline HbA1c and HbA1c change after treatment is not simply due to random error in baseline HbA1c being present in the change variable with subsequent regression to the mean.

### Regression to the mean does occur, although the effect size is small

**Fig 2:**
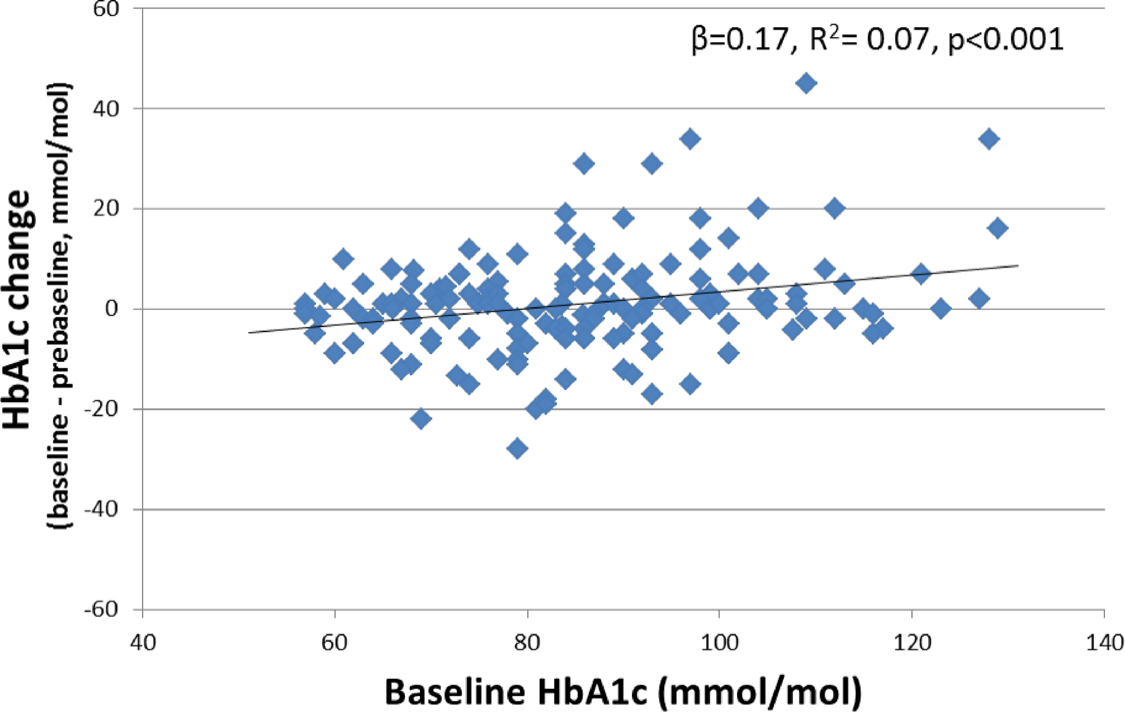
The relationship between baseline HbA1c and change from pre baseline to baseline HbA1c (GLP-1RA cohort). A positive HbA1c change denotes an increase between pre-baseline and baseline measurements.

Although the effect appears to be small, there is evidence of regression to the mean contributing to the greater HbA1c fall seen in those with higher baseline. **Fig 2**shows a plot of baseline HbA1c against change from pre-baseline to baseline HbA1c. This demonstrates that in those with high baseline HbA1c, the value has often increased from pre-baseline, in contrast to those with a low baseline where HbA1c has reduced from the previous value. The effect size appears to be small, explaining 7% of baseline HbA1c variation (baseline HbA1c against change from pre-baseline R^2^ = 0.07, β = 0.17 (0.07, 0.26), p<0.001).

### The association between baseline HbA1c and change after treatment persists when expressing change as a percentage of baseline

**Fig 3:**
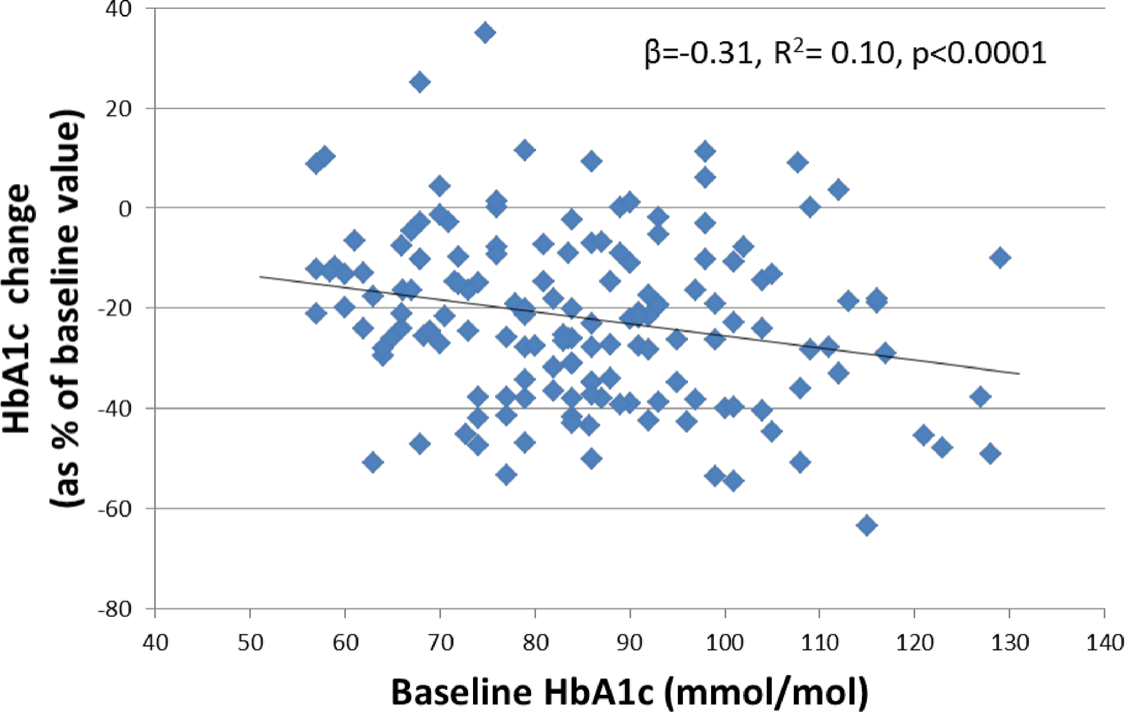
The relationship between Baseline HbA1c and post treatment HbA1c change after GLP-1RA therapy expressed as a percentage of baseline HbA1c.

When HbA1c change is expressed as a percentage of baseline the relationship between baseline HbA1c and treatment response persists (**Fig 3**); 10% of response variation is explained by baseline HbA1c with a 1mmol/mol increase in baseline HbA1c associated with an 0.3% greater HbA1c reduction post treatment (R^2^ = 0.10, linear regression β = -0.31 (95% CI -0.42, -0.20), p<0.001). This relationship persists using a separate baseline measurement (linear regression HbA1c change as percentage of baseline on pre-baseline HbA1c (R^2^ = 0.05, β = -0.24 (95% CI -0.39, -0.08), p = 0.004), suggesting expressing treatment response as a percentage of baseline may not fully remove the effect of baseline HbA1c.

### Adjustment for baseline HbA1c is necessary to avoid false associations between baseline HbA1c related covariates and response

**Fig 4:**
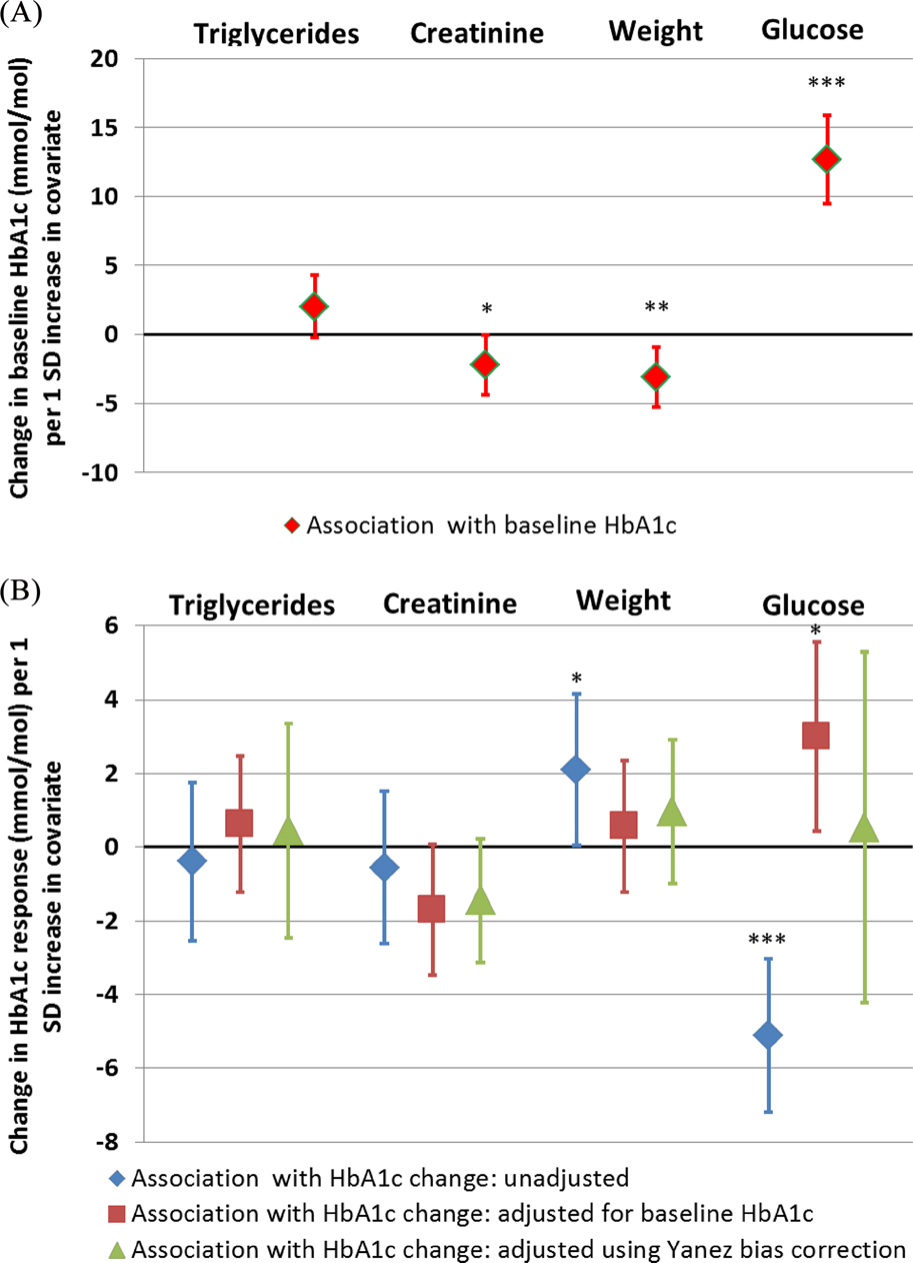
**The association between baseline covariates and A: baseline HbA1c, and B: glycaemic response to GLP-1RA therapy, with and without adjustment for baseline HbA1c and Yanez bias correction.** Effect sizes presented indicate A) baseline HbA1c or B) HbA1c response (6 months–baseline) difference (mmol/mol) for a 1 standard deviation increase in baseline covariate (linear regression β coefficient). A positive value represents higher baseline HbA1c with higher covariate (A) or reduced glycaemic response with higher baseline covariate (B). Error bars represent 95% confidence interval. N = 257. *p<0.05 **p<0.01 ***p<0.001.

**Fig 5:**
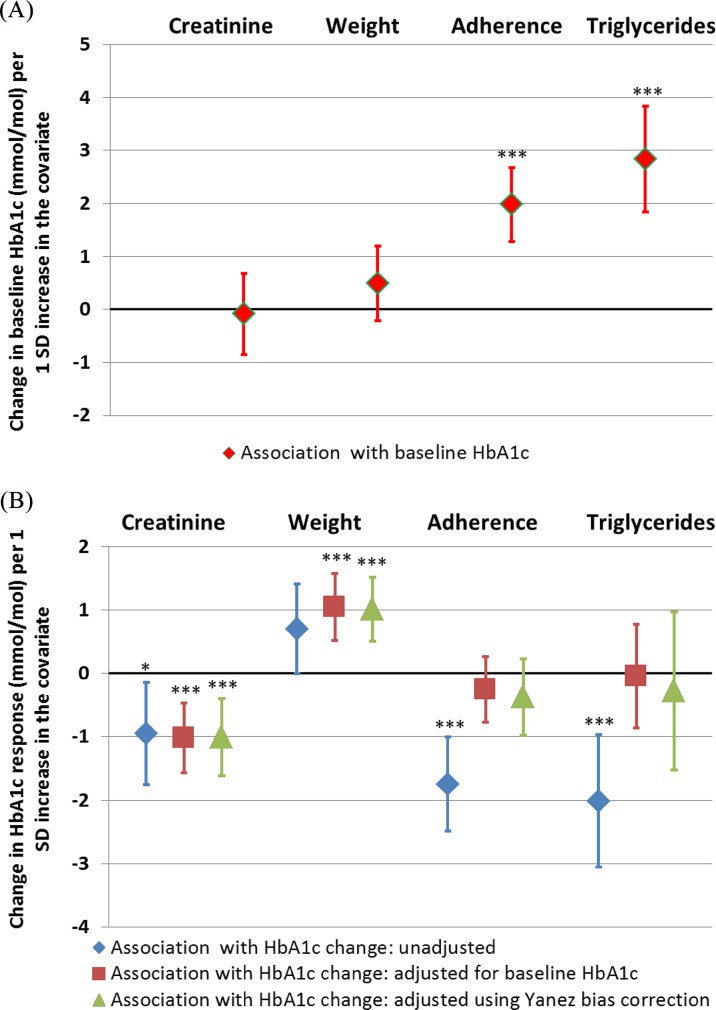
**The association between baseline covariates and A: baseline HbA1c, and B: glycaemic response to Sulfonylurea therapy, with and without adjustment for baseline HbA1c and Yanez bias correction.** Effect sizes presented indicate A) baseline HbA1c or B) HbA1c response (6 months–baseline) difference (mmol/mol) for a 1 standard deviation increase in baseline covariate (linear regression β coefficient). A positive value represents higher baseline HBA1c with higher covariate (A) or reduced glycaemic response with higher baseline covariate (B). Error bars represent 95% confidence interval. N = 1242 (triglycerides) to 2659 (adherence). *p<0.05 **p<0.01 ***p<0.001.

**Fig 4**(GLP-1R agonist cohort) and **Fig 5**(sulfonylurea cohort) show both the unadjusted and baseline-adjusted associations with post treatment HbA1c change for baseline characteristics with a range of associations with baseline HbA1c. Full data on these associations are given in **S2 Table and S3 Table.**

In an analysis not adjusted for baseline HbA1c in the GLP1RA cohort, weight and glucose are associated with treatment response, however these associations are absent or reduced after adjustment for baseline HbA1c. For all associations the effect sizes of baseline adjusted associations are closer to the bias corrected (Yanez) associations than unadjusted results, suggesting adjustment for baseline is appropriate.

In the Sulfonylurea cohort triglycerides and adherence were strongly associated with treatment response in unadjusted analysis however these factors were not associated with response after baseline adjustment, with or without bias correction. Again adjusted associations were closest to bias corrected results for all covariates.

### The Yanez bias correction does not substantially alter associations between covariates and treatment response unless a covariate is very highly correlated with baseline HbA1c

Adjusted results with and without the Yanez bias correction were similar for all associations with the exception of fasting glucose, which is very strongly associated with baseline HbA1c (r^2^ = 0.5), and therefore causes problems with multicollinearity. Adjusting the association between fasting glucose and response for baseline HbA1c without bias correction resulted in a weak association in the opposite direction to the unadjusted association, which was not present after bias correction.

### Expressing HbA1c change as a percentage of baseline provides only a partial correction for baseline HbA1c confounding

An alternative method of reducing confounding by baseline HbA1c associations is to express HbA1c change as a percentage of the baseline value. Using this method (without further baseline adjustment) fasting glucose remains associated with GLP1RA response (β = -2.8, p = 0.01, **S2 Table**) and both triglycerides and adherence remain associated with sulfonylurea response (triglycerides linear regression β = -0.35, p<0.1, adherence β = -4.0, p<0.0001, **S3 Table**).

## Discussion

This study demonstrates that the better glycaemic response seen in patients with higher baseline glycaemia is not mainly due to measurement error and regression to the mean, and that adjustment for baseline HbA1c is effective in reducing bias when assessing potential predictors of response to therapy. Adjustment for baseline HbA1c provides similar estimates to the bias-corrected results, closer than those obtained from the unadjusted models, suggesting this is the more appropriate analysis if repeated baseline HbA1c measures are not available.

While it has long been known that patients with higher glycaemia have a larger response to therapy to our knowledge this is the first study to assess whether this effect is due to regression to the mean and if baseline adjustment should be undertaken.

Strengths of this study include that we have been able to address these questions and show consistent findings in both a prospectively collected dataset and a large population dataset using two different glucose lowering agents. However, a limitation of this study is that it is observational and we have no placebo control group, which would allow formal analysis of treatment by baseline interaction. Further exploration in RCTs and using simulation studies would enable better estimation of the extent of regression to the mean in this setting. An additional limitation of this study is the lack of data on treatment change between pre-baseline and baseline HbA1c measurements in our GLP-1RA cohort. This means the differences between baseline and pre-baseline, and regression to the mean (as those with high pre baseline glycaemia would have been most likely to increase therapy) may be exaggerated. Had we been able to include only participants who did not change treatment between these two measurements the differences between baseline/pre-baseline response associations and the effect of bias correction may have been reduced. Likewise the observational nature of both our cohorts (participants receiving treatments as part of their usual clinical care) may have exaggerated regression to the mean, as patients are most likely to be prescribed a new treatment if HbA1c is higher than previous results. Therefore true baseline error and regression to the mean in an interventional study may be less than suggested by our results. The covariates we examined in this study were chosen on the basis of a range of association with baseline HbA1c to address a specific methodological question, this study is not intended to be a definitive study of predictors of treatment response which we have addressed in separate publications [16] [17]

While this study has only examined participants treated with GLP-1R agonists and sulfonylureas, it is likely these results would apply to all glucose lowering therapies: baseline HbA1c appears to influence response to all glucose lowering interventions [1–5] and the effect we are studying, the impact of random error in baseline HbA1c on the baseline response association, is unlikely to be treatment specific. Our results clearly demonstrate how false positive associations may arise from failure to adjust for baseline glycaemia, however it is also likely that true associations may be masked by negative confounding if baseline HbA1c is not adjusted for. We did not have clear examples of this effect within these cohorts.

The greatest difference in results between baseline adjusted results with and without bias correction in our cohorts were seen for fasting glucose, which was very highly associated with baseline HbA1c. This result should be interpreted with marked caution as these variables are very highly correlated and therefore beta coefficients may be erroneous due to multicollinearity [18]. However a plausible alternative explanation would be that adjusting for baseline is resulting in some overcorrection due to presence of regression to the mean, which may result in a weak association with the opposite direction of effect to an unadjusted result. Therefore we suggest weak associations with covariates calculated using glucose (such as HOMA) only present after baseline adjustment should be interpreted with caution, and that studies examining these variables consider repeated baseline HbA1c measures to address this potential bias. Further research including simulation studies and analysis of randomised placebo controlled trial data may be helpful to explore this further [19].

The findings of this study are important to the development of a stratified (or personalised) approach to the management of diabetes. Identifying biomarkers (including clinical characteristics, metabolites, proteins and genetic change) associated with response to therapy in diabetes may allow the targeting of specific therapies towards those most likely to benefit and have already demonstrated benefits in monogenic diabetes and in other fields such as oncology [20–23]. There is marked variation in whether current studies adjust results for baseline HbA1c, for example of 5 studies examining predictors of glycaemic response to GLP-1 receptor agonist therapy reported at the 2013 EASD meeting, 2 adjusted results for baseline and 3 did not [24]. Failure to adjust for baseline has the potential to introduce false associations or hide true associations where a biomarker is associated with baseline HbA1c. Our study suggests that baseline adjustment is likely to reduce error in this context. Our findings are applicable to the analysis of predictors of treatment response within an observational or interventional cohort. When analysing differential response between two therapies in a randomised controlled trial baseline HbA1c may be similar between groups at the same level of the predictive covariate due to randomisation, reducing but not necessarily removing the need for baseline adjustment [25].

In summary the relationship between baseline HbA1c and response to treatment is not predominantly due to regression to the mean; studies aiming to determine predictors of response to glucose lowering therapy should adjust for baseline HbA1c to reduce confounding by baseline glycaemia.

## Supporting Information

S1 Fig Overview of GLP-1RA cohort (PRIBA Study). (PPTX)

S1 Table Sulfonylurea cohort participants baseline characteristics (n = 2841). *n = 1809. (DOCX)

S2 Table **A: The effect of baseline HbA1c adjustment on the association between baseline covariates and HbA1c change after GLP-1RA therapy.** B = linear regression β coefficient, standardised for baseline covariates to represent HbA1c difference (in baseline HbA1c or change after treatment, mmol/mol) for a 1 standard deviation increase in baseline covariate. A positive β suggests a smaller HbA1c reduction with a higher value of the baseline covariate. * HbA1c change as a percentage of baseline HbA1c. **B: The association between baseline covariates and HbA1c change after GLP-1RA therapy, expressed as a percentage of baseline HbA1c.** β = linear regression β coefficient, standardised for baseline covariates to represent HbA1c response difference (as a percentage of baseline HbA1c) for a 1 standard deviation increase in baseline covariate. A positive β suggests a smaller HbA1c reduction with a higher value of the baseline covariate. Numbers in brackets represent the 95% confidence interval around β. * HbA1c Change as a percentage of baseline HbA1c.
(DOCX)

S3 Table **A: The effect of baseline HbA1c adjustment on the association between baseline covariates and HbA1c change after sulfonylurea therapy (GoDARTS).** β = linear regression β coefficient, standardised for baseline covariates to represent HbA1c response difference (as a percentage of baseline HbA1c) for a 1 standard deviation increase in baseline covariate. A positive β suggests a smaller HbA1c reduction with a higher value of the baseline covariate. Numbers in brackets represent the 95% confidence interval around β. **B: The association between baseline covariates and HbA1c change after Sulfonylurea therapy, expressed as a percentage of baseline HbA1c.** B = linear regression β coefficient, standardised for baseline covariates to represent HbA1c response difference (as a percentage of baseline HbA1c) for a 1 standard deviation increase in baseline covariate. A positive β suggests a smaller HbA1c reduction with a higher value of the baseline covariate.
(DOCX)

## Abbreviations

β: Regression coefficient, change in outcome variable for a 1 unit change in covariate
GLP-1RA: Glucagon like peptide-1receptor agonist
CPA: Clinical Pathology Accreditation
IFCC: International Federation of Clinical Chemistry and Laboratory Medicine
NHS: National Health Service (UK)
r: Correlation coefficient
R^2^: r squared
