# Supplementary material for: Should Studies of Diabetes Treatment Stratification Correct for Baseline HbA1c?
Source: PLoS One. 2016 Apr 6;11(4):e0152428. doi: 10.1371/journal.pone.0152428 (PMC4822872; doi:10.1371/journal.pone.0152428)
Supplement: S1 Fig — (PPTX) [file pone.0152428.s001.pptx]

## Slide 1
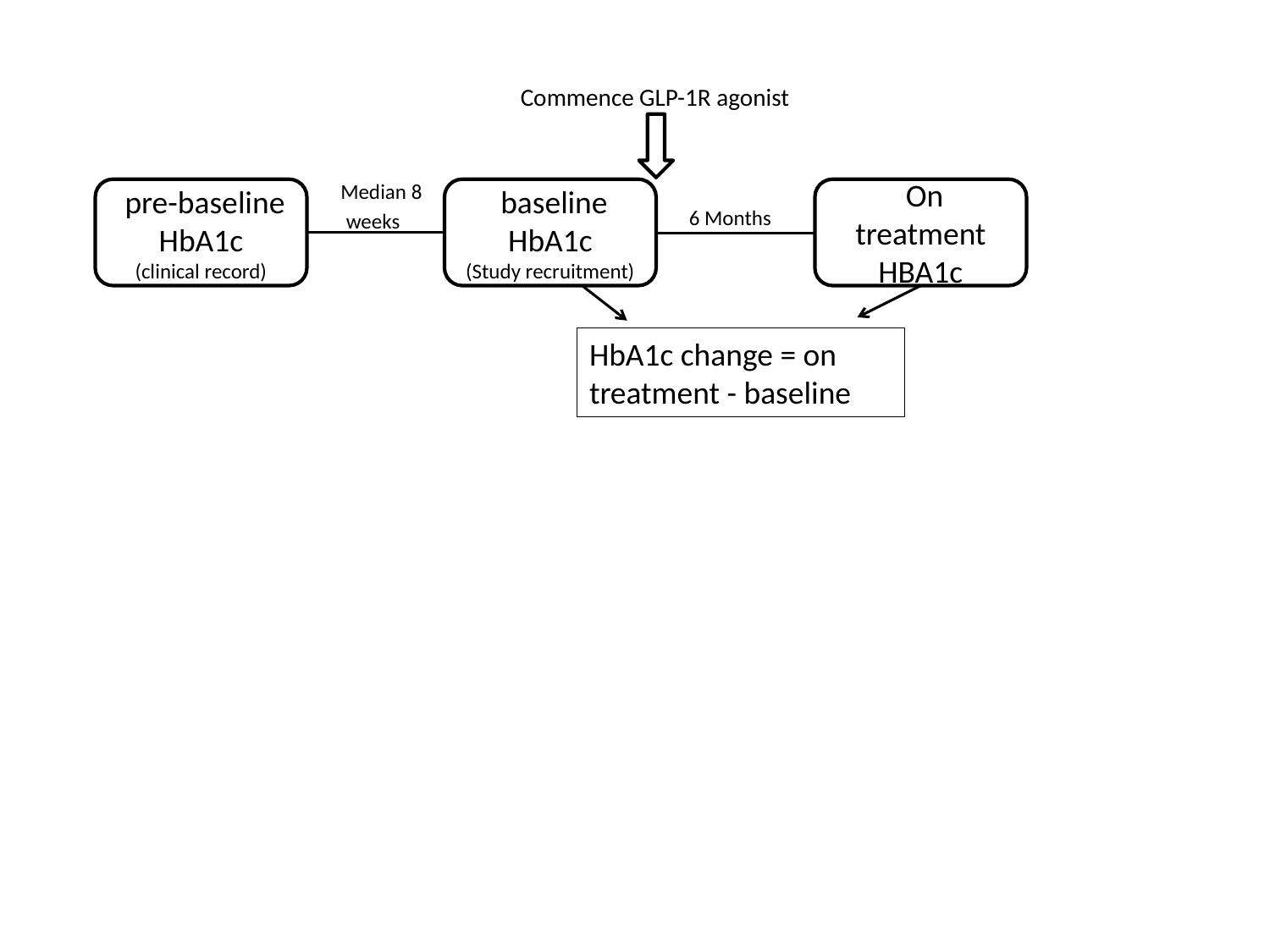

Commence GLP-1R agonist
Median 8 weeks
‘pre-baseline HbA1c
(clinical record)
‘baseline HbA1c
(Study recruitment)
‘On treatment HBA1c
 6 Months
HbA1c change = on treatment - baseline
