# Supplementary material for: Should Studies of Diabetes Treatment Stratification Correct for Baseline HbA1c?
Source: PLoS One. 2016 Apr 6;11(4):e0152428. doi: 10.1371/journal.pone.0152428 (PMC4822872; doi:10.1371/journal.pone.0152428)
Supplement: S1 Table — (DOCX) [file pone.0152428.s002.docx]

**Table S1: Sulphonylurea cohort participants baseline characteristics (n=2841)**

| **Baseline characteristics** | **Median (IQR) or %** |
| --- | --- |
| Baseline HbA1c (mmol/mol) | 71.6 (62.1-84.7) |
| Pre-baseline HbA1c (mmol/mol)* | 70.1 (61.7, 82.5) |
| Time from pre-baseline to baseline HbA1c (days)* | 132 (83-190) |
| HbA1c change (6months – 0 months, mmol/mol) | -15,3 |
| % Male | 56 |
| Age (years) | 64 (57-72) |
| Diabetes duration (years) | 4 (2-7) |
| BMI (kg/m2) | 29.8 (26.9-33.8) |
| Weight (kg) | 86.9 (73.7-96.5) |
| Triglycerides (mmol/L) | 2.3 (1.6-3.4) |
| Creatinine (umol/L) | 85 (73-98) |
