# Supplementary material for: Should Studies of Diabetes Treatment Stratification Correct for Baseline HbA1c?
Source: PLoS One. 2016 Apr 6;11(4):e0152428. doi: 10.1371/journal.pone.0152428 (PMC4822872; doi:10.1371/journal.pone.0152428)
Supplement: S2 Table — A: The effect of baseline HbA1c adjustment on the association between baseline covariates and HbA1c change after GLP-1RA therapy. B = linear regression β coefficient, standardised for baseline covariates to represent HbA1c difference (in baseline HbA1c or change after treatment, mmol/mol) for a 1 standard deviation increase in baseline covariate. A positive β suggests a smaller HbA1c reduction with a higher value of the baseline covariate. * HbA1c change as a percentage of baseline HbA1c. B: The association between baseline covariates and HbA1c change after GLP-1RA therapy, expressed as a percentage of baseline HbA1c. β = linear regression β coefficient, standardised for baseline covariates to represent HbA1c response difference (as a percentage of baseline HbA1c) for a 1 standard deviation increase in baseline covariate. A positive β suggests a smaller HbA1c reduction with a higher value of the baseline covariate. Numbers in brackets represent the 95% confidence interval around β. * HbA1c Change as a percentage of baseline HbA1c. (DOCX) [file pone.0152428.s003.docx]

**Table S2 A: The effect of baseline HbA1c adjustment on the association between baseline covariates and HbA1c change after GLP-1RA therapy.** B = linear regression β coefficient, standardised for baseline covariates to represent HbA1c difference (in baseline HbA1c or change after treatment, mmol/mol) for a 1 standard deviation increase in baseline covariate. A positive β suggests a smaller HbA1c reduction with a higher value of the baseline covariate.

* HbA1c change as a percentage of baseline HbA1c

| **Association (linear regression)** | **Triglyceride (mmol/L)** | **Creatinine (umol/L)** | **Weight (kg)** | **Fasting glucose (mmol/L)** |
| --- | --- | --- | --- | --- |
| **n** | 239 | 254 | 257 | 229 |
| **Association with baseline HbA1c** | β=2.02  (-0.22, 4.3)  p=0.08 | β=-2.2  (-4.4, -0.12)  p=0.04 | β=-3.1  (-5.3, -0.96)  p=0.005 | β =12.7  (11.1, 14.3)  p=<0.0001) |
| **1. Association with HbA1c change: Unadjusted** | β =-0.39  (-2.5, 1.8)  p=0.72 | β =-0.55  (-2.6, 1.5)  p=0.6 | β =2.1  (0.07, 4.2)  p=0.04 | β =-5.1  (-7.1, -3.0)  p=<0.0001 |
| **2. Association with HbA1c change: Adjusted by baseline HbA1c** | β =0.63  (-1.2, 2.5)  p=0.5 | β =-1.7  (-3.5, 0.07)  p=0.06 | β =0.57  (-1.2, 2.4)  p=0.5 | β =3.0  (0.43, 5.5)  p=0.02 |
| **3. Association with HbA1c change:**  **Adjusted using Yanez bias correction*** | β =0.45  (-2.7, 3.1)  p=0.53 | β =-1.45  (-3.2, 0.14)  p=0.08 | β =0.96  (-0.89, 3.0)  p=0.3 | β =0.53  (-4.4, 5.1)  p=0.8 |

**Table S2 B: The association between baseline covariates and HbA1c change after GLP-1RA therapy, expressed as a percentage of baseline HbA1c.** β = linear regression β coefficient, standardised for baseline covariates to represent HbA1c response difference (as a percentage of baseline HbA1c) for a 1 standard deviation increase in baseline covariate. A positive β suggests a smaller HbA1c reduction with a higher value of the baseline covariate. Numbers in brackets represent the 95% confidence interval around β.

* HbA1c Change as a percentage of baseline HbA1c

| **Association (linear regression)** | **Triglyceride (mmol/L)** | **Creatinine (umol/L)** | **Weight (kg)** | **Fasting glucose (mmol/L)** |
| --- | --- | --- | --- | --- |
| **n** | 239 | 254 | 257 | 229 |
| **Association with percentage HbA1c change*** | β=0.09  (-2.1, 2.3)  p=0.9 | β=-0.73  (-2.8, 1.4)  p=0.5 | β=1.5  (-0.61, 3.6)  p=0.16 | β=-2.8  (-4.9, 0.65)  p=0.01 |
