# Supplementary material for: Should Studies of Diabetes Treatment Stratification Correct for Baseline HbA1c?
Source: PLoS One. 2016 Apr 6;11(4):e0152428. doi: 10.1371/journal.pone.0152428 (PMC4822872; doi:10.1371/journal.pone.0152428)
Supplement: S3 Table — A: The effect of baseline HbA1c adjustment on the association between baseline covariates and HbA1c change after sulfonylurea therapy (GoDARTS). β = linear regression β coefficient, standardised for baseline covariates to represent HbA1c response difference (as a percentage of baseline HbA1c) for a 1 standard deviation increase in baseline covariate. A positive β suggests a smaller HbA1c reduction with a higher value of the baseline covariate. Numbers in brackets represent the 95% confidence interval around β. B: The association between baseline covariates and HbA1c change after Sulfonylurea therapy, expressed as a percentage of baseline HbA1c. B = linear regression β coefficient, standardised for baseline covariates to represent HbA1c response difference (as a percentage of baseline HbA1c) for a 1 standard deviation increase in baseline covariate. A positive β suggests a smaller HbA1c reduction with a higher value of the baseline covariate. (DOCX) [file pone.0152428.s004.docx]

**Table S3 A: The effect of baseline HbA1c adjustment on the association between baseline covariates and HbA1c change after sulphonylurea therapy (GoDARTS).**  β = linear regression β coefficient, standardised for baseline covariates to represent HbA1c response difference (as a percentage of baseline HbA1c) for a 1 standard deviation increase in baseline covariate. A positive β suggests a smaller HbA1c reduction with a higher value of the baseline covariate. Numbers in brackets represent the 95% confidence interval around β.

| **Association (linear regression)** | **Creatinine (umol/L)** | **Weight (kg)** | **Adherence (%)** | **Triglycerides**  **(mmol/l)** |
| --- | --- | --- | --- | --- |
| **Sample size** | 2315 | 2376 | 2659 | 1242 |
| **Association with baseline HbA1c** | β=-0.09  (-0.86, 0.69)  p=0.8 | β=0.49  (-0.22, 1.20)  p=0.2 | β=1.98  (1.27, 2.69)  p<0.0001 | β=2.84  (1.84, 3.95)  p<0.0001 |
| **1. Association with HbA1c change: Unadjusted** | β=-0.95  (-1.75, -0.14)  p=0.02 | β=0.70  (-0.029, 1.44)  p=0.05 | β=-1.75  (-2.48, -1.01)  p<0.0001 | β=-2.02  (-3.06, -0.97 )  p=0.0001 |
| **2. Association with HbA1c change: Adjusted by baseline HbA1c** | β=-1.02  (-1.56, -0.46)  p=0.0002 | β=1.06  (0.53, 1.58)  p<0.0001 | β=-0.25  (-0.77, 0.26)  p=0.3 | β=-0.041  (-0.83, 0.74)  p=0.9 |
| **3. Association with**  **HbA1c change:**  **Adjusted by Yanez**  **bias correction*** | β=-1.01  (-1.62,-0.40)  p=0.0008 | β=1.015  (0.51, 1.52)  p=0.0002 | β=-0.37  (-0.98, 0.23)  p=0.2 | β=-0.27  (-1.25, 1.25)  p=0.7 |

**Table S3 B: The association between baseline covariates and HbA1c change after Sulphonylurea therapy, expressed as a percentage of baseline HbA1c.** B = linear regression β coefficient, standardised for baseline covariates to represent HbA1c response difference (as a percentage of baseline HbA1c) for a 1 standard deviation increase in baseline covariate. A positive β suggests a smaller HbA1c reduction with a higher value of the baseline covariate.

| **Association (linear regression)** | **Creatinine (umol/L)** | **Weight (kg)** | **Adherence (%)** | **Triglycerides (mmol/l)** |
| --- | --- | --- | --- | --- |
| **Sample size** | 2315 | 2376 | 2659 | 1242 |
| **Association with percentage HbA1c change*** | β=-0.63  (-1.48, 0.22)  p=0.14 | β=0.61  (-0.19, 1.42)  p=0.13 | β=-1.4  (-2.17, -0.60)  p=0.0004 | β=-1.17  (-2.29, -0.05)  p=0.03 |
